# Glacier ablation and temperature indexed melt models in the Nepalese Himalaya

**DOI:** 10.1038/s41598-019-41657-5

**Published:** 2019-03-27

**Authors:** Maxime Litt, Joseph Shea, Patrick Wagnon, Jakob Steiner, Inka Koch, Emmy Stigter, Walter Immerzeel

**Affiliations:** 1 Physical Geography, Faculty of Geosciences, Utrecht University, Utrecht, The Netherlands; 2 International Center for Integrated Mountain Development, Kathmandu, Nepal; 3 Geography Program, University of Northern British Columbia, Prince George, Canada; 4 Univ. Grenoble-Alpes, CNRS, IRD, Grenoble INP, F-38000, Grenoble, France

## Abstract

Temperature index (TI) models are convenient for modelling glacier ablation since they require only a few input variables and rely on simple empirical relations. The approach is generally assumed to be reliable at lower elevations (below 3500 m above sea level, a.s.l) where air temperature (*T*_a_) relates well to the energy inputs driving melt. We question this approach in High Mountain Asia (HMA). We study *in-situ* meteorological drivers of glacial ablation at two sites in central Nepal, between 2013 and 2017, using data from six automatic weather stations (AWS). During the monsoon, surface melt dominates ablation processes at lower elevations (between 4950 and 5380 m a.s.l.). As net shortwave radiation (*SW*_net_) is the main energy input at the glacier surface, albedo (*α*) and cloudiness play key roles while being highly variable in space and time. For these cases only, ablation can be calculated with a TI model, or with an Enhanced TI (ETI) model that includes a shortwave radiation (*SW*) scheme and site specific ablation factors. In the ablation zone during other seasons and during all seasons in the accumulation zone, sublimation and other wind-driven ablation processes also contribute to mass loss, and remain unresolved with TI or ETI methods.

## Introduction

In High Mountain Asia (HMA), present and future glacier down wasting and retreat will lead to changes in seasonal and spatial patterns of meltwater contributions to streamflow^1,2^. In highly glacierised catchments, changes in meltwater inputs will considerably affect the timing and magnitude of river discharge. Given the large population densities in the region and the strong socio-economic dependence on streamflow for irrigation, industry, and power generation, reliable estimates of future glacier mass change are critical.

Ablation represents the ensemble of processes that lead  to ice and snow mass loss. It includes melt, sublimation or evaporation at the surface, as well as wind-driven transport and sublimation of blowing snow^3^. By implicitly assuming that surface melt is the dominant ablation term, hydrological and glaciological models often use temperature-index (TI) or enhanced temperature-index (ETI) approaches to quantify total ablation. Such models conveniently rely only on near-surface air temperature, as well as a degree-day factor (*TF*_TI_) which depends on the state of the surface i.e., ice or snow, and assume that melt is a linear function of the sum of the difference between the daily mean air temperature *T*_a_ and an air temperature threshold (*T*_TI_)^4,5^. This relationship is generally well-established at lower altitudes. The ETI model builds on the TI approach by including the contribution of the daily mean net incoming shortwave radiation (*SW*_net_) multiplied by a shortwave radiation factor (*SRF*, kg day^−1^ W^−1 ^^6^). At high elevation glaciers, such as those found throughout High Mountain Asia (HMA), incoming shortwave radiation (*SW*_inc_) is the dominant energy input^7^ and the SEB relates only partly to daily mean *T*_a_, so numerous studies use ETI in place of TI. Estimates of albedo (*α*) and *SW*_inc_ are required for an ETI approach, and  *SW*_inc_ can be approximated  as theoretical potential shortwave incoming at the top of the atmosphere. Atmospheric transmissivity, cloudiness and *α* can be estimated from empirical models calibrated on *T*_a_ and relative humidity (RH) data (see Methods for a detailed description).

However, surface melt is more suitably modeled with the net surface energy balance (SEB), which accounts for the energy entering the ice or snow surface from above (downward, positive) and that leaving it (negative), upwards to the air, or downwards to the deeper layers, by heat conduction and transport. TI and ETI calibrations depend on the driving components of the SEB. These differ over time, latitude, and elevation. At mid-latitude and low-elevation sites, the TI is reliable since *SW*_inc_, net longwave radiation (*LW*_net_) and the turbulent flux of sensible heat (*H*) are well correlated with *T*_a_ and have a strong control on glacial and snow melt. At high elevation sites, the links between glacial melt and *T*_a_ or *SW* and the TI (or ETI) relations have rarely been validated^8^. Studies of glacier SEB in the HMA^7,9–14^, and other high-altitude regions^15–17^ remain scarce (Table 1). On top of net shortwave radiation (*SW*_net_), they demonstrate the importance of *LW*_net_ as a loss and indicate relatively small turbulent heat fluxes, which are generally positive for sensible heat (*H*) and negative for latent heat (*LE*).

**Table 1:** Daily mean contributions from net shortwave and longwave radiation and turbulent fluxes from various energy balance studies in High Mountain Asia.

	Study	Position	Elevation, m a.s.l	Period	*SW* [Wm^−2^]	*LW* [Wm^−2^]	*H*+*LE* [Wm^−2^]
Baltoro Glacier	Collier *et al*.^13^	Karakoram	4022–5875	from 25 Jun 2004 to 31 Aug 2004	*SW*_inc_ = 374.3	*LW*_inc_ = 220.4	*H* = 9.5
					*SW*_out_ = −186.4	*LW*_out_ = −306.1	*LE* = −13.9
					*SW*_net_ = 187.9	*LW*_net_ = −85.7	
Chhota Shigri	Azam *et al*.^7^	Indian Himalayas	4670	from 8 Jul 2013 to 5 Sep 2013	*SW*_*inc*_ *= 248*	*LW*_*inc*_ *= 302*	*H* = 31
					*SW*_*out*_ *= -47*	*LW*_*out*_ *= −315*	*LE* = 11
					*SW*_net_ = 202	*LW*_net_ = −14	
AX010	Kayastha *et al*.^39^	Nepal Himalayas	5245	from 25 May 1978 to 25 Sep 1978	*SW*_net_ + *LW*_net_ = 55		*H* = 8
							*LE* = 3
Zhadang Glacier	Molg *et al*.^11^	Southeast Tibetan Plateau	5660	from Apr 2009 to Sep 2011	*SW*_inc_ = 260.4	*LW*_inc_ = 200.9	*H* = −3.4
					*SW*_out_ = −187.7	*LW*_out_ = −262.2	*LE* = −10.9
					*SW*_net_ = 63.1	*SW*_net_ = −61.7	
Parlung N0. 04	Yang *et al*.^40^	Southeast Tibetan Plateau	4800	from 25 Jun 2009 to 21 Aug 2009	*SW*_net_ = 164	*SW*_net_ = −31	*H* = 22
							*LE* = −4
Laohugu No. 12	Sun *et al*.^12^	Western Qilian mountains	4550	from 1 Jun 2011 to 30 Sep 2011	*SW*_net_ = 126	*SW*_net_ = −45	*H* = 6.5
							*LE* = −12.8
Purogangri	Huintjes *et al*.^14^	Central Tibetan Plateau	5350–6370	from 1 Sep 2001 to 1 Sep 2011 (summer values)	*SW*_inc_ = 292	*LW*_inc_ = 234	*H* = 26
					*SW*_out_ = −253	*LW*_out_ = −268	*LE* = −24
					*SW*_net_ = 39	*LW*_net_ = −34	

**Figure 1:**
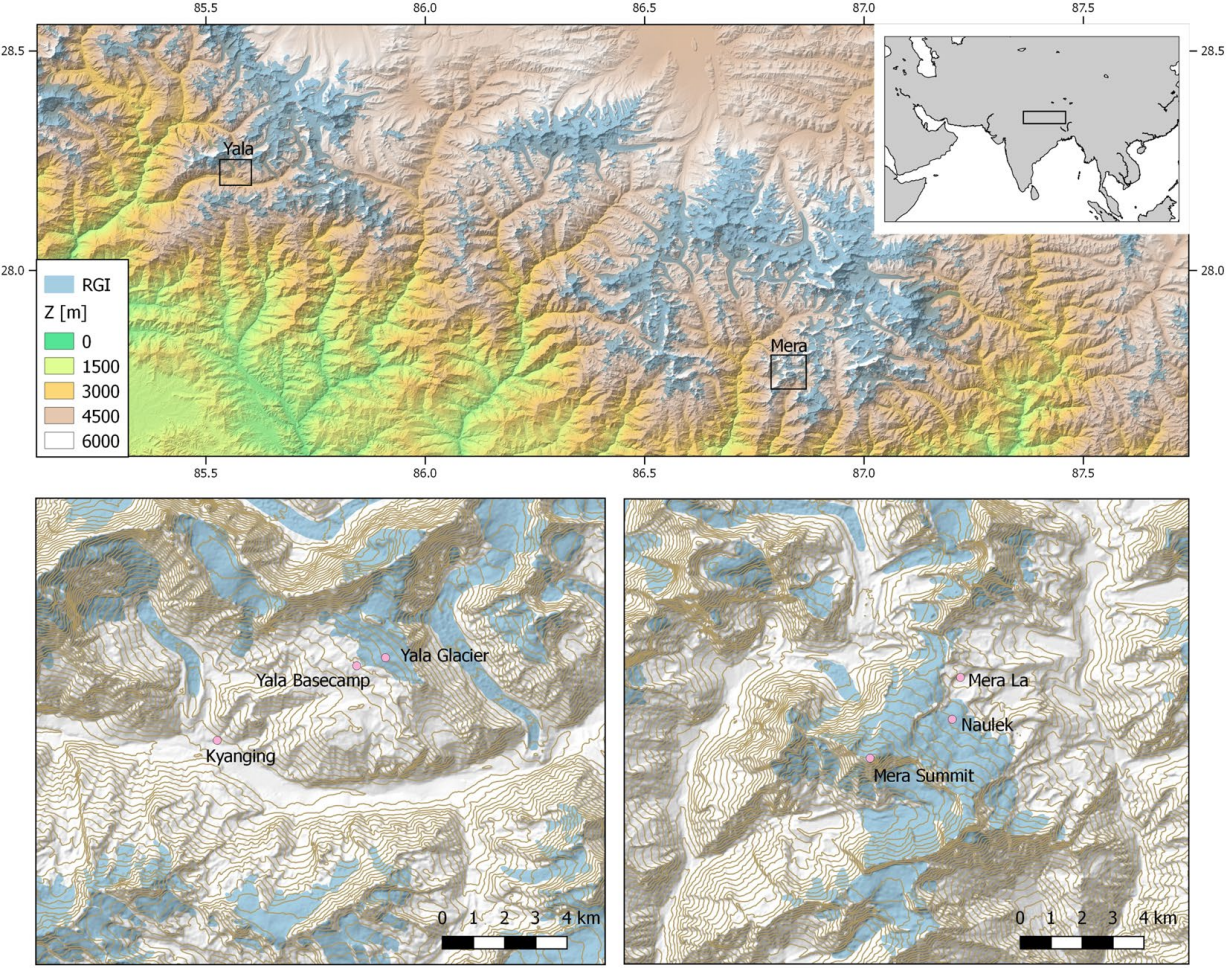
Study area map with elevation (Z), location of measurement sites (circles), and RGI5.0 glacier extents (light blue).

**Figure 2:**
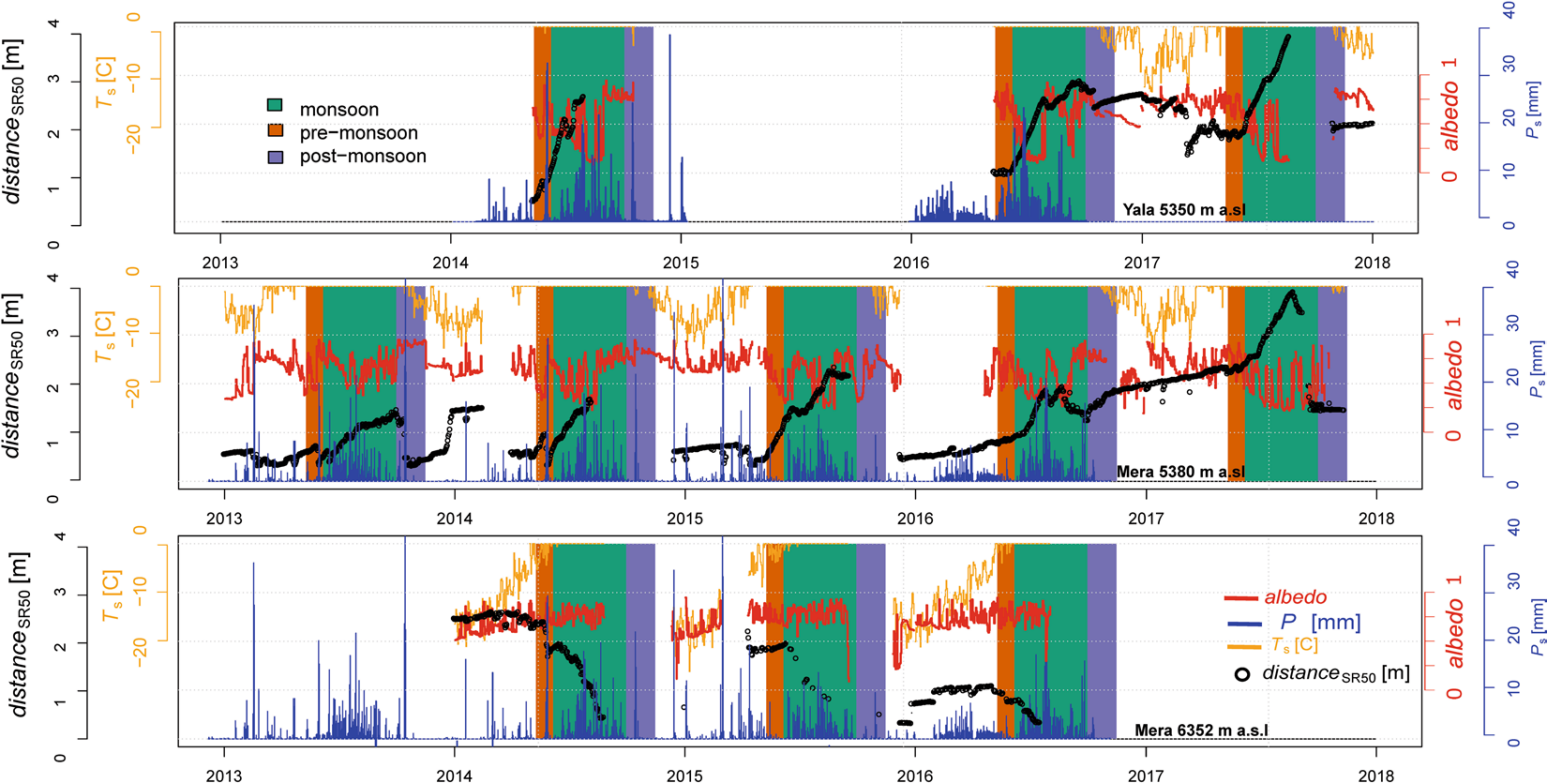
Summary of meteorological and surface lowering data from the two ablation zone sites (Yala Glacier, 5350 m a.s.l. and Mera Glacier, 5380 m a.s.l.) and the one accumulation zone site (Mera Glacier, 6352 m a.s.l.) between 2013 and 2017.

**Figure 3:**
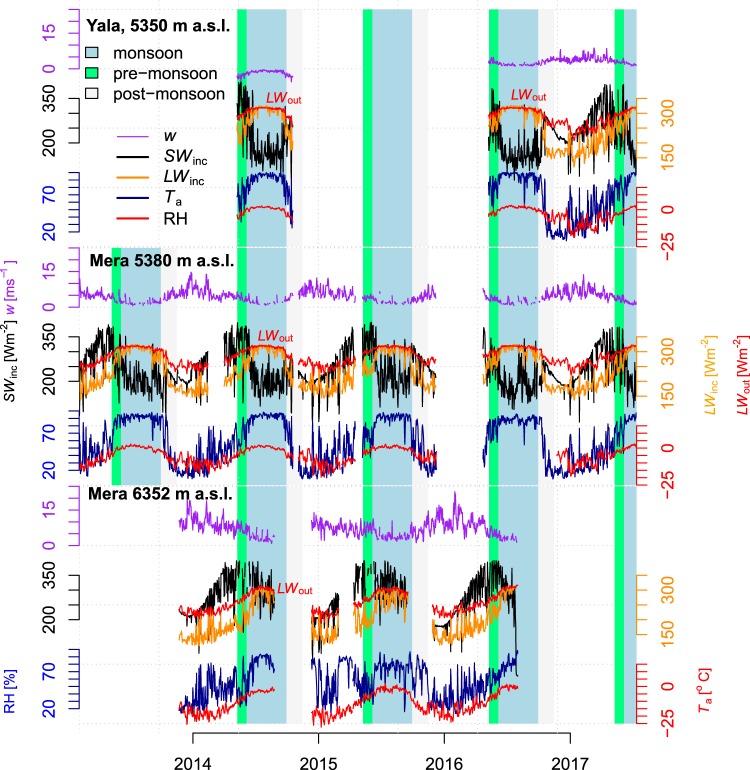
Daily averages of hourly measurements from the meteorological variables at the three on-glacier automatic weather stations: Mean incoming shortwave (black) and longwave (orange) radiation, mean air temperature (red), mean wind speed (purple) and relative humidity (blue). Mera Glacier, 6352 m a.s.l., was buried in snow on 28^th^ of July 2016 resulting into extreme or no average variables.

**Figure 4:**
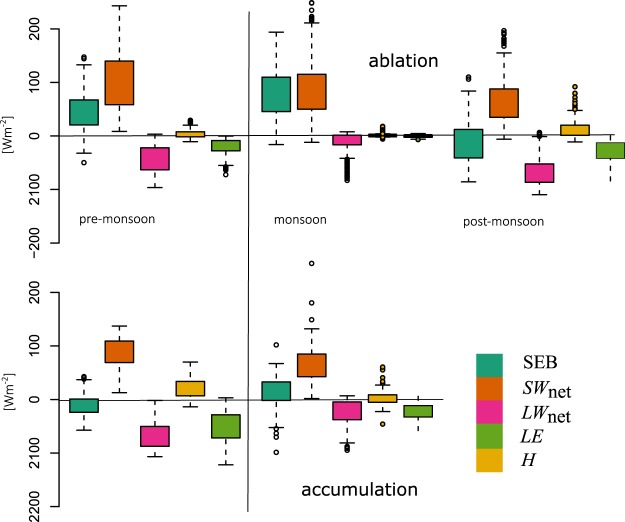
Boxplot summaries of mean daily surface energy balance components calculated from all available data, classified by season and grouped into ablation (upper panel) and accumulation (lower panel) sites. Each box upper (resp. lower) edge is drawn at the third quartile (resp. first) of the variable distribution, and whiskers provide the same value plus (resp. minus) the interquartile range. Values outside the whiskers are provided by the dots.

**Figure 5:**
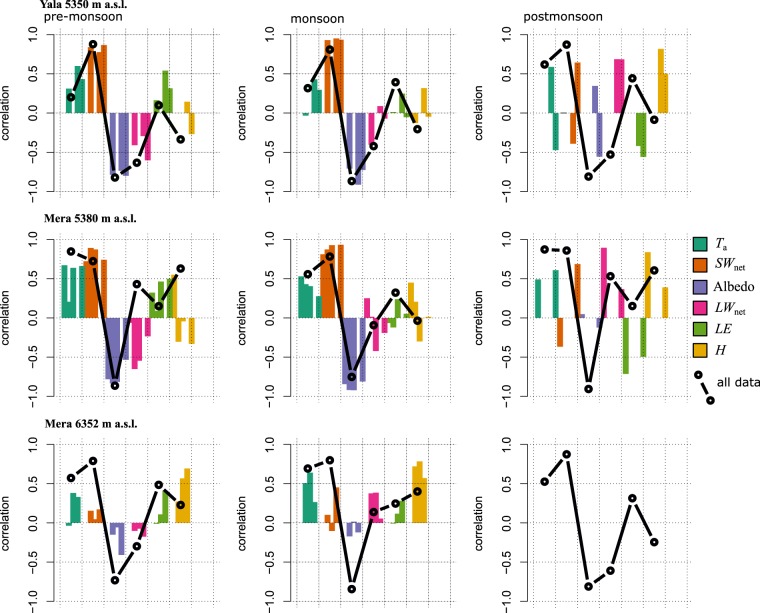
Values of the Pearson’s correlation coefficient between the daily mean SEB and daily mean air temperature (R(*T*_a_, SEB)), the daily mean SEB and daily mean SEB components (R(*x*, SEB)) and the daily mean SEB and *α* (R(*α*, SEB)), during the three defined seasons. Each bar represents a season’s value for one given year. The circles joined with the black line indicate the value of the correlation coefficients obtained when combining the data from all the years for a given season and site.

**Figure 6:**
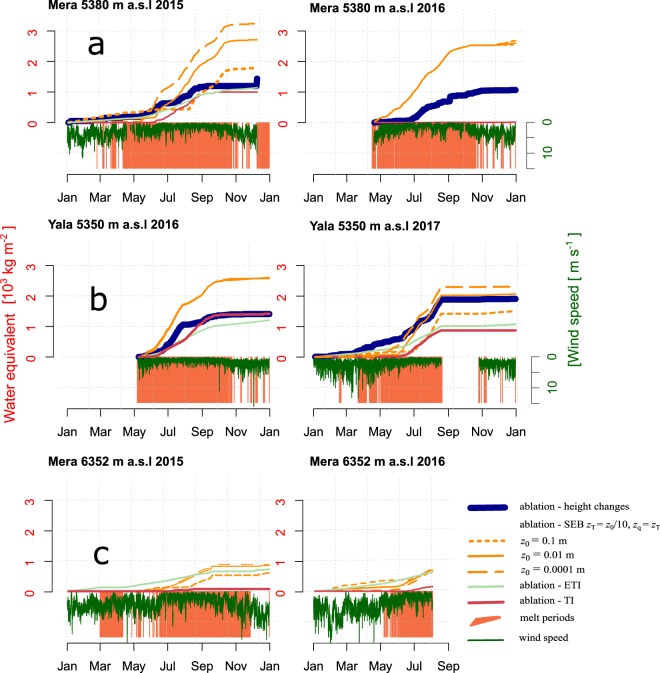
Cumulated ablation calculated with the surface lowering measurements (thick blue line), with the surface energy balance for changing *z*_0_ values (orange dashed and continuous lines), with the TI (red line) and ETI (clear blue line) with one fixed set of factors. The hourly wind speed is shown upside down (green curve). Periods of surface melt (*T*_s_ = 0) are highlighted in orange. Results from Mera Glacier, 5380 m a.s.l in 2014 and 2017 (**a**) from Yala Glacier, 5350 m a.s.l., in 2014, 2016 (**b**) and Mera Glacier, 6352 m a.s.l, in 2015 and 2016 (**c**).

Sublimation, wind-driven snow redistribution, and blowing snow sublimation processes must be accounted for as part of net ablation^3,18^. Such processes have previously been shown to be important for glacier ablation in the region^18,19^. These processes are not directly linked to *T*_a_ or *SW*, and calibrations of TI and ETI upon observed ablation are not necessarily consistent in time and space when melt is not the only important contributor^4,20,21^. The numerical simulation of these additional ablation processes is necessary and possible through physical models but requires knowledge of many physical variables. Their use is thus severely constrained by *in-situ* data availability. That is why the TI and ETI models are preferably employed in HMA^22,23^ to quantify melt or even total ablation. Here, we question their validity, and we provide guidelines for future use of these models in the region. Our study relies on *in-situ* measurements of glacier surface lowering and energy balance obtained in the Nepalese Himalaya (Fig. 1). In the Results, we describe our observations of surface height and *α* changes, surface temperature (*T*_s_) on the glacier, and the other surface characteristics (Fig. 2), together with the observed meteorological conditions (Fig. 3). We then show statistics for the SEB and its components (Fig. 4). Subsequently we present correlations between daily SEB, its components and on-glacier *T*_a_ or *α* (Fig. 5, see also Table S2). This permits us to assess the significance of key individual meteorological and surface variables in the SEB, and the relevance of TI and ETI parameters for surface ablation at different sites and for the different seasons. In the Discussion, we calibrate TI and ETI models to the observed surface ablation as derived from glacier surface height measurements and discuss the temporal and spatial consistency of their parameters (Fig. 6, see Methods for details). We analyse the statistical relations between *T*_a_, SEB, glacial melt and ablation in terms of physical processes. This provides a critical view of ablation modelling with TI and ETI models in HMA environments. In the Conclusions we propose guidelines for future studies. Details about the Methods are provided in the final section.

## Results

### Observed climatology and surface height changes

The Nepalese Himalayas are heavily influenced by the South Asian monsoon^24^, which brings most of the annual precipitation to the region between June and September. Following this annual pattern we defined 3 seasons with identical start and end dates each year. This is optimized for data availability, maximizing duration of overlapping time ranges with simultaneous observations of surface characteristics and meteorology (Figs 1 and 2). The pre-monsoon period, characterized by increasing humidity and temperatures^23^ starts 10 May and finishes 5 June. The monsoon is defined as 6 June–30 September^25^, and the post-monsoon period extends from 1 October to 15 November. We do not discuss the winter season in detail. Data availability and measurement details are found in Table 2.

**Table 2:** Summary of meteorological measurements used in this study.

Station	Elevation m a.s.l.	Time range	Variables	Sensor Precision
Mera Glacier ablation zone	5380	27 Nov 2013–12 Dec 2015	*T*_a_, RH	Vaisala HMP155 ±0.2 °C, ±2%
			*w*	Young 05103-5 ±0.3 m s^−1^
			*SW*, *LW*,	Kipp&Zonen CNR4 ±3%
			inst. height	Campbell SR50 ±0.01 m
		21 Apr 2016–1 Jan 2017	*T*_a_, RH	Vaisala HMP155 ±0.2 °C, ±2%
			*w*	Young 05103-5 ±0.3 m s^−1^
			*SW*, *LW*,	Kipp&Zonen CNR4 ±3%
			inst. height	Campbell SR50 ±0.01 m
			*(T*_*a*_ from 21 Nov 2016)	
Mera-La	5350	11 Nov 2013–13 Nov 2016	*T*_a_, RH	Vaisala HMP155 ±0.2 °C, ±2%,
			*w*	Young 05103-5 ±0.3 m s^−1^
			*SW*, *LW*, (from 3 Mar 2014),	Kipp&Zonen CNR4 ±3%
			Pa (from 21 Nov 2013).	
				Campbell Sci. CS100 ±0.3 hPa
Pluviometer Pyramid	5035	6 Dec 2012–27 Apr 2016	Precipitation	Geonor T-200B ±0.1 mm
Pluviometer Pheriche	4260	27 Apr 2016–5 Nov 2016	Precipitation	Geonor T-200B ±0.1 mm
			(gap from 16 Feb 2014 to 11 Apr 2014)	
Mera Glacier accumulation zone	6352 (near summit)	20 Nov 2013–26 Aug 2014	*T*_a_, RH	Vaisala HMP155 ±0.2 °C, ±2%
			*w*	Young 05103-5 ±0.3 m s^−1^
			*SW*, *LW*,	Kipp&Zonen CNR4 ±3%
		10 Dec 2014–2 Aug 2016 Station buried in snow on 28^th^ of July	Inst. Height (measured between 11 Apr 2015–6 Sep 2015 and 9 Dec 2015–2 Aug 2016)	Campbell SR50 ±0.01 m
Yala Glacier ablation zone	5350	5 May 2014–19 July 2014	*T*_a_, RH	Rotronic HC2S3 ±0.1 °C, ± 0.8%
			*w*	Young 05103L-45 ±0.3 m s^−1^
			*SW*, *LW*,	Kipp&Zonen CNR4 ±3%
			Pa,	Vaisala PTB330 ±0.20 hPa
			Snow depth	Jenoptik SHM30
		5 May 2016–18 Aug 2016 and 26 Oct 2016–1 Jan 2017	*T*_a_, RH	Rotronic HC2S3 ±0.1 °C, ± 0.8%
			*w*	Young 05103L-45 ±0.3 m s^−1^
			*SW*, *LW*,	Kipp&Zonen CNR4 ±3%
			Pa,	Campbell Sci. CS106 ±1.5 mb
			Snow depth	Campbell Sci. SR50 ±0.01 m
Yala Glacier base camp	4950	5 May 2014–14 Dec 2014	*T*_a_, RH	Rotronic HC2S3 ±0.1 °C, ± 0.8%
			*w*	Young 05103L-45 ±0.3 m s^−1^
			*SW*, *LW*,	Kipp&Zonen CNR4 ±3%
			Pa	Vaisala PTB330 ±0.20 hPa
			precipitation	OTT Pluvio 2 ±0.1 mm
		27 Oct 2016–1 Jan 2017	*T*_a_, RH	Rotronic HC2S3 ±0.1 °C, ±0.8%
			*w*	Young 05103L-45 ±0.3 m s^−1^
			*SW*, *LW*,	Kipp&Zonen CNR4 ±3%
			Pa	Campbell Sci. CS106 ±1.5 mb
			precipitation	OTT Pluvio 2 ±0.1 mm

Annual surface lowering of up to 2 m was observed at the lowest ablation zone sites of Yala and Mera Glaciers (Fig. 2). In the ablation zone, this height change can be attributed to mass loss through mainly surface melt, as daily maximum surface temperatures (*T*_s_) reach 0 °C (Fig. 2., see Methods for the calculation of *T*_s_). However, surface lowering also occurs when daily maximum *T*_s_ is below 0 °C. This indicates that processes such as snow compaction, wind-driven erosion or sublimation must be occurring, as suggested by previous studies over glaciers in the HMA^18,19,26^. Also, surface lowering rates were variable in time (Fig. 2). Precipitation events resulted in fresh snow deposition, as indicated by increases in surface height: these events also increased *α*, and were more frequent during monsoon than during other seasons. During the monsoon, the high-elevation accumulation zone site on Mera Glacier experienced both surface height gains and losses. Surface melt is a possible contributor to this surface lowering, since the *T*_s_ occasionally reached 0 °C (Fig. 2). However, observations of densified ice layers inside the snowpack at this site indicate that water refroze in the lower layers: the glacier remains cold at this elevation^18^ and ablation therefore likely occurred through other processes.

The large scale atmospheric forcing was similar over both glaciers, as reflected by the meteorological measurements which evolved synchronously^23^ (Fig. 3). At all sites, incoming shortwave radiation (*SW*_inc_) was greatest at the beginning of June^27^, with an average daily value of 304 W m^−2^ for all pre-monsoon periods between 2010–2015^28^. However, *SW*_inc_ quickly decreased to an annual minimum at the summer solstice. This is due to increasing cloud cover starting from the end of May, as humid air masses are transported northwards by the South Asian monsoon, which is active until September in central Nepal^24^. In October, *SW*_inc_ increased rapidly as the monsoon clouds and humidity retreated and clear skies returned to the region. *SW*_inc_ then followed a standard seasonal pattern with a second minimum at the winter solstice. At the Mera accumulation zone site, *SW*_inc_ was generally more intense than at the ablation zone sites, and this is likely due to Sun’s radiation   reaching ground after having crossed less atmospheric layers.

At all sites, high elevation and cold clear skies favor low values of incoming longwave radiation (*LW*_inc_), especially during winter^17^ (Fig. 3). Since clouds emit large amounts of thermal radiation^29,30^ cloudiness is strongly linked to *LW*_inc_ at high altitudes. Cloudiness increased progressively through spring to reach a plateau in summer, as shown by the *LW*_inc_ increase. At all sites, daily mean *T*_a_ rose synchronously with net longwave radiation (*LW*_net_), starting from a minimum at the end of January and reaching a plateau of its highest values at the beginning of June each year. In the ablation zones of both glaciers daily mean *T*_a_ varied between −10 °C and 0 °C, but *T*_a_ rarely exceeded 0 °C as any excess energy was converted to snow or ice melt. Following the end of monsoon, *T*_a_ and *LW*_inc_ decreased rapidly, though *T*_a_ showed large variations during autumn and winter. At Mera Glacier accumulation zone AWS (6352 m a.s.l), located about a thousand meters higher than the ablation zone sites (Fig. 1, Table 2), mean daily *T*_a_ values are consistently lower, and temperatures below −25 °C were observed in winter. The mean daily wind speed (*w*) remained below 10 m s^−1^ at 5350 m a.s.l. on Yala Glacier, while at 5380 m a.s.l on Mera Glacier, mean daily *w* greater than 15 m s^−1^ were observed. The accumulation zone site at Mera Glacier (6352 m a.s.l.) experienced the greatest mean daily *w* (>20 m s^−1^). At all sites the maximum wind speed was observed during winter and pre-monsoon, and is related to westerly disturbances and south to north shifts in the position of the jet stream. Daily mean *w* was the lowest during monsoon (~2 m s^−1^). Daily mean relative humidity (RH) was similar at all sites. Its variability was greatest during the pre-monsoon, and lowest during the monsoon. In all years, RH increased progressively from its minimum (~20%) in January to a plateau of 90–100% between June and September. RH then decreased abruptly in October, back to values around its minima.

### Ablation Zone Surface Energy Balance and Mass Loss

Below, we present the statistics of the SEB components. The temporal correlations of total surface energy balance (SEB) with its components *x* and with *T*_a_, *α*, are noted as R(*x*, SEB). Partial correlations are noted R(*y*, SEB)_*x*_ (where *y* can be any variable, see Methods). The partial correlation of *y* with SEB indicates whether or not the correlation of *y* with SEB is due to a strong correlation of *y* with *x*. SEBs were strongly site- and season-dependent (Fig. 4). On average, pre-monsoon SEBs were positive at ablation zone sites. There the main energy input during pre-monsoon was +*SW*_net_ (daily mean ~+100 Wm^−2^). *LW*_net_ was a loss of energy of about −50 Wm^−2^, similar to that observed on tropical glaciers in South America^17,31^, in a similar climatic setting. Sensible turbulent fluxes (*H*) were positive, but latent heat fluxes (*LE*) were negative. On average, there was a slight net energy loss due to turbulent energy fluxes (*H* + *LE* ~ −15 Wm^−2^). Positive net SEBs and surface temperatures of 0 °C (Fig. 2) in the ablation zone suggest that the observed surface lowering was mainly driven by melt.

Variations in the SEB between sites and years were negatively correlated with *LW*_net_ (R(*LW*_net_, SEB) = −0.52, Fig. 5, Table 3) and poorly or not at all correlated with the turbulent fluxes (R(*H*, SEB) = 0.12, R(*LE*, SEB) < 0.0). We assume that melt controls ablation when *T*_s_ = 0 °C, and directly convert positive SEB into surface melt. We obtain an average melt rate of −41 kg m^−2^ day^−1^ (Table 4). However, in the ablation zone mass loss also occurred through evaporation/sublimation, as indicated by the negative values of *LE* which correspond to an average mass loss of −3 kg m^−2^ day^−1^ over all the pre-monsoon periods (Table 4). During monsoon, the SEB increased (Figs 3, 4), with net daily energy gains greater than 100 Wm^−2^. Substantial increases in *LW*_inc_ due to monsoon cloud cover were largely responsible for the SEB gains, as monsoon *LW*_net_ was only slightly negative. Surface ablation was large (−75 kg m^−2^ day^−1^ on average) and mainly due to melt as *T*_s_ was 0 °C. Turbulent fluxes were low or null. Differences in the SEB between the two ablation sites were likely due to varying *SW*_net_ (R(*SW*_*net*_, SEB) = 0.90). This suggests that a fast alternation between snow and ice surfaces exerts a strong control on the SEB through *α* changes (R(*α*, SEB) = −0.80). Monsoon SEBs are weakly correlated with *LW*_net_ (R(*LW*_net_, SEB) = 0.15), which signifies that cloudiness and higher longwave radiation totals are relatively stable. Nevertheless, the *SW*_net_ controls on the SEB are rather linked to changes in *LW*_net_ than to changes in *α* (R(*SW*_net_, SEB)_*LW*net_ = 0.19 against R(*SW*_net_, SEB)_*a*_ < 0.01, Table S2). During post-monsoon at the ablation zone sites, clear sky conditions and high *α* (>0.8) after monsoon snowfalls reduce *SW*_net_ (often below 100 Wm^−2^), and large losses of energy occurred through large negative *LW*_net_ (<−50 Wm^−2^). The controls of *LW*_inc_ over SEB are large^32^, reaching R(*LW*_inc_, SEB) = 0.66 at Yala ablation site (Table 3). Net turbulent fluxes were also negative, and dominated by negative latent heat fluxes, favored by intense wind. Small ablation totals were likely due to sublimation as *T*_s_ was mostly negative during the post-monsoon (Fig. 2), and net SEB was often negative.

**Table 3:** Correlation coefficients between surface energy balance components, air temperature and albedo, cumulating all available data.

R(*x*, SEB)		*T*_a_	*SW*_net_	albedo	*LW*_net_	*LW*_inc_	*LW*_out_	*H*	*LE*
Yala, 5350 m a.s.l.	pre-monsoon	0.35	0.86	−0.82	−0.52	−0.45	−0.01	0.12	0.00
(ablation zone)	monsoon	0.53	0.93	−0.83	−0.08	−0.04	0.18	0.11	0.08
	post-monsoon	0.15	−0.04	−0.03	0.64	0.66	0.62	−0.46	0.70
Mera, 5380 m a.s.l.	pre-monsoon	0.36	0.82	−0.80	−0.44	−0.49	0.21	0.30	−0.07
(ablation zone)	monsoon	0.55	0.90	−0.86	−0.16	−0.15	0.21	0.09	0.00
	post-monsoon	NA	0.74	−0.14	0.35	0.62	0.72	−0.66	0.64
Mera, 6352 m a.s.l.	pre-monsoon	−0.14	−0.16	0.01	0.21	0.29	0.27	−0.00	0.72
(accumulation zone)	monsoon	0.24	0.01	−0.06	0.37	0.42	0.44	0.05	0.75
	post-monsoon	NA	NA	NA	NA	NA	NA	NA	NA

**Table 4:** Summary of the governing ablation processes and their relative contribution to the ablation (assuming no wind erosion).

		Pre-monsoon	Monsoon	Post-monsoon
Ablation zone:	Melt	94%, −41 kg m^−2^ day^−1^	79%, −75 kg m^−2^ day^−1^	65%, −5 kg m^−2^ day^−1^
	Sublimation	6%, −3 kg m^−2^ day^−1^	21%, −25 kg m^−2^ day^−1^	35%, −2 kg m^−2^ day^−1^
	Number of days	Mera 109,	Mera 299,	Mera 81,
		Yala 64	Yala 164	Yala 37
Accumulation zone	Melt	0% 0 kg m^−2^ day^−1^	7% −9 kg m^−2^ day^−1^	No data
	Sublimation	100% −7 kg m^−2^ day^−1^	93% −12 kg m^−2^ day^−1^	
	Number of days	Mera 42	Mera 64	

### Accumulation Zone Surface Energy Balance and Mass Loss

The accumulation zone SEB is notably different from ablation zone sites during the main melt periods (pre-monsoon and monsoon). At the Mera Glacier accumulation zone site, *SW*_net_ was the main source of energy in the pre-monsoon. However, SEB remained negative throughout the pre-monsoon and surface melt was zero as *T*_s_ < 0 °C (Fig. 2). As this site (6352 m a.s.l.) is higher than the other sites, *LW*_inc_ was lower and *LW*_net_ was more negative (*LW*_net_ ~ −100 Wm^−2^). Turbulent fluxes were more intense than at the lower elevation sites due to higher wind speeds, and RH values were generally lower (Fig. 3). As a result, net turbulent fluxes were negative (−25 Wm^−2^ on average), and reached minimum values of −80 Wm^−2^. Observed surface lowering was due mainly to wind-driven erosion, compaction, or sublimation. During the monsoon, *SW*_net_ at the Mera Glacier accumulation zone site was lower than during pre-monsoon. Reduced atmospheric transmissivity due to clouds and humidity resulted in decreased *SW*_inc_ (50 to 100 Wm^−2^), while frequent fresh snow falls (Fig. 2) increased *α* and thus *SW*_*out*_. The cloud emission raised *LW*_inc_, but not as much as at the lower sites. *H* + *LE* was still negative and variable through the monsoon. Accumulation zone net SEB was thus lower than observed at ablation zone sites, and more variable. Our calculations indicate that sublimation as inferred from the latent heat fluxes (−12 kg m^−2^ day^−1^, Table 4) is a larger contributor to mass loss than melt (−9 kg m^−2^ day^−1^). While wind-driven ablation processes must be a significant factor for surface lowering and is not captured by our set up, enhanced sublimation rates for wind-blown snow^18^ suggest that our surface sublimation rate is a conservative estimate.

### Air temperature, surface energy balance, melt and ablation

Correlations between *T*_a_, SEB, and SEB components provide insights into the mechanisms driving the variation of the melt. At the ablation zone sites on Yala and Mera Glaciers during pre-monsoon, variations in the mean daily SEB were highly correlated to changes in mean daily *SW*_net_ (R(*SW*_net_, *SEB*) ~0.6 to 0.9, Fig. 5), and less but still significantly, to daily mean *T*_a_ (R(*T*_a_, SEB) ~0.3 to 0.5). The contribution of the statistical relation existing between *T*_a_ and *SW*_net_ to the overall R(*T*_a_, SEB) (noted R(*T*_a_, SEB)_*SW*net_, Methods), was between 35% and 42% (Table S3). Mean daily *T*_a_ was highly correlated to *SW*_net_ as days with high insolation totals were warmer. This, in turn, enhances the entire SEB through increased *LW*_inc_ and *H*. During the monsoon, *T*_a_ is similarly related to the SEB, but *SW*_net_ is better correlated to the SEB than during pre-monsoon. We observe a high negative correlation of *α* with the SEB at ablation zone sites (Fig. 5), for which daily mean *T*_a_ was a poor indicator. In the accumulation zone at Mera Glacier (6352 m a.s.l.), during pre-monsoon, *T*_a_ and *SW*_net_ variations related much less to the SEB than at the low elevation, ablation zone sites (Fig. 5). Overall, individual radiation terms did not explain the small SEB changes well. Similarly, during the monsoon, *T*_a_ as well as *SW*_net_ variations are less correlated to the SEB (R ~ 0.3) than at the lower elevation sites. Permanent snow cover at the high elevation site drives high *α*, and relatively low variability in *SW*_net_. *LW*_net_ also exhibited a weak link with the SEB. However, the net radiation related well to the net SEB. Changes in the SEB related well to changes in *H* (R(*H*, SEB) ~0.49) and *LE* (R(*LE*, SEB) ~0.70), significantly more than in the ablation zone, as winds were more intense (Figs 3, 6). There was not enough data for a post-monsoon analysis.

## Discussion

The performance of the ETI and TI using only one set of parameters for modelling the observed ablation at different sites and periods is limited (Fig. 6). The lack of consistency in TI and ETI parameters between sites and years (Table S4) is similarly problematic. For many cases, the calibration procedure even fails to optimize the parameters. Ablation modeled with SEB can diverge from the observations, but a suitable value for surface roughness can solve the issue (Fig. 6). When wind speed is high the SEB is especially sensitive to a change in roughness length, further highlighting the importance of wind related processes. At the ablation sites, the TI and ETI factors calibrated are highly variable, and depend on the season, site and year (Table S4). At ablation zone sites, pre-monsoon melt totals (Fig. 6, Table 4) are low and driven by *SW*_net_. Factors are calibrated in order to reproduce observed ablation, even though daily mean *T*_a_ is not a good indicator of the pre-monsoon SEB (i.e. Results, Fig. 5, Table 3). Using individual factors for snow and ice directly includes the effect of changing surface *α* but results in increased factor variability. ETI shortwave radiation factors (*SRF*) are more stable from site to site and from year to year (Table S4). Ablation factors remain slightly variable, probably due to the presence of ablation processes such as surface sublimation, wind-driven surface erosion and snow redistribution. These processes do not relate well to *T*_a_, contributing to changes of the TI or the ETI factors. TI factors for snow show low variability during the monsoon season, though this is likely due to low snow melt totals. Conversely, calibrated ice ablation factors range from 1 to 45 kg m^−2^ day^−1^ °C^−1^. At ablation zone sites, calibrated ice ablation factors are more variable and the calculated ablation is thus more sensitive to the parameter choices than during pre-monsoon.

Empirical ablation models need to accurately simulate the response of melt to large variations in *SW*_net_. These changes primarily occur due to rapid and large changes in *α* caused by frequent precipitation events that deposit thin layers of snow on the ice surface. There is an additional inter-site and inter-year variability of *TF*_TI_ and *TF*_ETI_. Outgoing *LW* remains constant since *T*_s_ is always 0 during this melt-dominated period, so variations of cloudiness and *LW*_out_ must be partly responsible for inter-year variability in calibrated ablation factors. Changes in the surface sublimation or wind-driven erosion and snow redistribution, which are not well related to *T*_a_, could also contribute to ablation factor variability. The whole assessment is also strongly limited by the point-scale nature of our measurement set-up. In the accumulation zone of Mera Glacier, some very limited surface lowering was observed (Fig. 2) but was poorly captured by the TI, the ETI, and the ablation derived from SEB (*A*_SEB_, Fig. 6, see also Table S3 in Supplementary Material). The actual lowering can be explained only by surface compaction, densification or by wind-driven erosion and sublimation. Even though *SW*_net_ is the main input for *A*_SEB_ it does not vary much since *α* remains high. Furthermore, small increases in *SW*_net_ only occur under clear skies, when *LW*_net_ is a large loss and *w* is stronger, which both favor low *T*_a_. *LW*_net_ is more variable at the accumulation zone site than at lower elevations, and it drives changes in *T*_a_.

At high elevations, the SEB relates poorly to *T*_a_ and *SW*_net_, and furthermore represents only one part of mass loss. Therefore, the use of TI/ETI approaches to model ablation in this context is questionable. We conclude that for the presented sites, ablation models should (1) account for highly variable changes in albedo and cloudiness that affect melt rates, and (2) accommodate additional ablation processes such as wind-driven snow erosion and snow redistribution, as well as blowing snow sublimation. Improved and expanded observational studies at high elevation sites are thus necessary^26^. Models that can incorporate such processes (e.g. MESO-NH^33,34^ or COSMO-WRF^35^) should be explored with suitable test data. The correlation study conducted here shows that *α* has a strong control on the SEB. However, it is the change from snow to ice cover that exerts this strong control, since in the accumulation zone where surface remains snow covered, the correlation is much lower than in the ablation zone where surface transitions are more frequent. This highlights the additional importance of correctly characterizing the snow/rain temperature threshold. We note that *α* at the onset of monsoon is relevant for the total ablation over the season, and this is likely controlled by the amount of fallen spring snow.

## Conclusions and Perspectives

In this study, meteorological controls on glacier ablation in High Mountain Asia (HMA) were studied using *in-situ* meteorological data collected at two glaciers in Nepal. We estimated the amount of mass loss due to surface melt and other ablation processes. The main variables controlling surface ablation were identified, and parameter values for a temperature-index (TI) and an enhanced temperature-index (ETI) model were provided. Modelling skill and parameters were assessed with respect to the observed meteorology at ablation zone and accumulation zone sites. Both models, calibrated for one season, perform well in the ablation zone during pre-monsoon and monsoon, since ablation is dominated by melt for those settings. A reliable estimate of surface *α* is nevertheless essential, since surface energy balance (SEB) changes are controlled by the changes in net shortwave radiation. Due to site and season-specific changes in the climatic drivers of ablation, the factors found for modeling ablation with a TI or an ETI are only transferable from one site to the other or from year to another year for what we define as the pre-monsoon period. During monsoon, surface melt (intense at times) dominates net ablation, though it has a site-dependent rate. TI and ETI calibrations are influenced by inter-annual changes in cloudiness. Also, site-specific changes in the snow-rain temperature point, in fresh-snow density, or in evaporation and sublimation rates, prevent the transferability of calibrated parameters to another site. During post-monsoon, melt remains limited and other ablation processes such as snow erosion by wind or sublimation are important but not statistically related to air temperature. This leads to the conclusion that a real alternative^33–35^ to TI and ETI should be developed for these cases. Existing models that are able to account for these processes^32,33^^,^^35^ are currently still computationally expensive. Surface lowering can be observed on some days at the high elevation site (Mera Glacier, 6352 m a.s.l.) during pre-monsoon and monsoon. This lowering is unlikely to be driven by melt, and is rather due to sublimation or other wind-driven processes, especially during pre-monsoon. Some ablation through surface melt may be occurring during monsoon but it remains weak and meltwater is typically refrozen in the snowpack below. The TI and ETI approaches cannot predict this weak high elevation monsoon melt, especially if calibrated in the ablation zone, as at high elevations air temperatures are below the necessary threshold for melt onset. The daily mean *T*_a_ relates poorly to melt totals as the *SEB* is positive mainly when there are clouds and when sublimation is reduced, due to lower wind speeds. The overall dependence of melt on *α* highlights the importance of collecting accurate *α* measurements and developing *α* models specific for high elevation sites. Accurate quantification of snowfall amounts and timing are consequently critical, and high elevation studies on the rain-snow threshold temperature are required.

## Methods

### Study sites

We used detailed on-glacier measurements between 2014 and 2017 on Yala Glacier (28.81 N, 85.84E, 1.61 km^2^) in the Langtang Valley and on Mera Glacier (27.7 N, 86.9E, 5.1 km^2^) in the Khumbu region. Measurements are made at accumulation and ablation zone sites at elevations ranging between 5350 and 6352 m a.s.l., (Fig. 1, Table 2). In addition, we use measurements from two AWS fixed on bedrock near the terminus of each glacier (Fig. 1, Table 2), and data from nearby pluviometers in the Khumbu valley. Mera Glacier, located approximately 30 km south of Mt. Everest, straddles the Hinku Valley and Hunku Valley in Central Nepal. There are two branches of the same glacier in the ablation zone, known as Naulek Glacier and Mera Glacier. Mera Glacier ranges in elevation from 4940 to 6420 m a.s.l.^18^, faces north in the accumulation area and south-east in the ablation area of Naulek branch. The monitoring program on Mera Glacier was initiated in 2007^18,28^. Yala Glacier is a small south-west facing glacier located approximately 135 km west of Mera Glacier in Langtang Valley, Nepal. Yala Glacier ranges in elevation from 5128 to 5749 m a.s.l.^23,36^.

### Data

On-glacier meteorological measurements were obtained from ‘floating’ stations (Yala Glacier, May to October 2014) and stations drilled into the ice surface (Yala Glacier, May 2016 to October 2017). At Mera Glacier, 5380 m a.s.l., ablation zone site, an AWS operated continuously between November 2012 and November 2016, while the AWS on Mera Glacier, 6352 m a.s.l. (near summit), was operational intermittently between November 2013 and July 2016 due to difficult meteorological conditions from 26 August 2014. Continuous meteorological data from Mera Glacier, 6352 m a.s.l., are only available from November 2013 to 26 August 2014, from December 2014 to March 2015, from April 2015 until August 2015 then December 2015 to 2 August 2016 (Table 1). The Mera Glacier, 5380 m a.s.l., ablation zone station, was drilled in ice until March 2014, and after was on a free standing tripod. Mera Glacier, 6352 m a.s.l., station was drilled in ice. Additionally, at both glacier sites in the Langtang and Everest areas, an AWS was permanently operated nearby the glacier tongue on rocky and ice-free surfaces, at 5350 m a.s.l. on Mera La for the Naulek-Mera site and at 5090 m a.s.l at Yala Glacier base camp. All stations measure the 4 components of the surface radiative balance, air temperature and humidity, wind speed and direction, and surface height changes resulting a priori from ice or from snow deposition or ablation processes. Precipitation is recorded at Yala Glacier base camp AWS. Precipitation was also measured in the Khumbu region at the Pyramide site and at Pheriche (Table 1, Fig. 1). Atmospheric pressure was measured intermittently at the different AWS locations. Details of the settings and data availability can be found in Table 2.

### Surface energy balance and statistics

We defined the hourly surface energy balance (SEB) in W m^−2^ as:

1 $${\rm{S}}{\rm{E}}{\rm{B}}=(S{W}_{inc}+S{W}_{out}+L{W}_{inc}+L{W}_{out}+H+LE)$$

Where *SW*_inc_, *SW*_out_, *LW*_inc_ and *LW*_out_ are the incoming and outgoing shortwave and longwave radiation, *H* and *LE* are the turbulent fluxes of sensible and latent heat fluxes, respectively. The individual SEB components were measured or calculated from the AWS data. The radiative fluxes were given by the radiometers. The turbulent fluxes *H* and *LE* were derived using the so-called bulk-aerodynamic method^37^, and using the glacier AWS measurements: barometric pressure (*P*_a_), wind speed (*w*), air temperature (*T*_a_) and relative humidity (RH) and snow/ice surface temperature *T*_s_ calculated from the measured surface longwave emission, assuming snow and ice emissivity (*ε*) was equal to 0.99. The aerodynamic (*z*_0_), thermal (*z*_0t_) and humidity (*z*_0q_) roughness lengths were set constant to 0.01 m, 0.001 m and 0.001 m, respectively. We used stability corrections as in Litt *et al*.^37^. The values of the aerodynamic roughness length can be seen as high but are based on Stigter *et al*.^26^, who calibrated them on Yala Glacier so that bulk fluxes fit fluxes as evaluated from an eddy-covariance tower.

In order to provide insights on the meteorological processes governing SEB, we calculated the Pearson correlation coefficient (R) between (i) each daily mean component of the SEB and the daily mean SEB, (ii) of the temperature with the daily mean SEB and (iii) with all the daily mean SEB components. We then (iv) calculate the contribution of each SEB term *x* to the correlation between the SEB and the daily mean *T*_a_ and (v) to the correlation between the SEB and the shortwave radiation, as:

2 $${\rm{R}}{({T}_{{\rm{a}}},{\rm{SEB}})}_{x}=\frac{{\sigma }_{x}}{{\sigma }_{SEB}}{\rm{R}}({T}_{a},x)$$

3 $${\rm{R}}{(S{W}_{net},{\rm{SEB}})}_{x}=\frac{{\sigma }_{x}}{{\sigma }_{SEB}}{\rm{R}}(S{W}_{{\rm{net}}},x)$$

These partial correlations indicate the degree of variability of variable *x*, relating to *T*_a_ (Eq. 2) or to the *SW*_net_ (Eq. 3), and also to the net SEB. If (2) or (3) are high for a given *x* and R(*T*_a_, SEB) is high, this indicated that the *x* variable explained well the SEB variability, in relation to *T*_a_.

### Ablation models

Since we did not measure the fluxes below the surface, we used hourly surface temperature as a proxy for melt occurrence. When *T*_s_ reached 0 °C once after the winter, hourly negative SEB values were accumulated as a “cold storage” term. This cold storage had to be compensated for and as soon as the SEB was positive and surface temperature was 0 °C we converted the energy to hourly melt. The sum of the melt derived from the SEB plus the ablation due to sublimation and evaporation derived from *LE* was called *A*_SEB_ in kg m^−2^. This was validated against the observed ablation (*A*_obs_). It was obtained by converting the measured daily surface height changes to water equivalent, using a density (*ρ*, kg m^−3^) parameterization based on the observed *α*, inspired by that used in^38^ and tuned to the observations such as:

4 $$\alpha  < 0.2,\rho =850\,{\rm{kg}}\,{{\rm{m}}}^{-3}({\rm{ice}})$$

5 $$\alpha  > 0.2,\rho =680\to 5\,{\rm{kg}}\,{{\rm{m}}}^{-3}({\rm{firn}})$$

### Temperature index (TI) and enhanced temperature index (ETI) Models

To account for specificities of the site and season, we calibrated a unique set of the TI and ETI parameters. We discuss its ability to model observed ablation at other sites, comparing the calibrated parameters to each other (Table S4). For the TI we have, if the daily mean of *T*_a_ is larger than *T*_TI_:

6 $${A}_{{\rm{TI}}}=T{F}_{{\rm{TI}}}(\overline{{T}_{{\rm{a}}}}-{T}_{{\rm{TI}}})$$

The overbar indicates daily mean. *TF*_TI_ kg m^−2^ °C ^−1^ day^−1^ is given a different value when *α* is higher than 0.4 (snow-melt factor) or lower than 0.4 (ice-melt factor). For the ETI model we have, if the daily mean *T*_a_ is larger than *T*_ETI_:

7 $${A}_{{\rm{ETI}}}=T{F}_{{\rm{ETI}}}(\overline{{T}_{{\rm{a}}}}-{T}_{{\rm{ETI}}})+SRF.S{W}_{{\rm{inc}}}(1-\alpha )$$

The *TF*_ETI_ is set the same for all surface states, and *A*_ETI_ stands for the daily ablation (m we) derived from the *SRF* (m we W^−1^ m^2^ day^−1^) and the daily mean *SW*_inc_. We calibrate the two parameters *TF*_TI_ for snow and for ice, the *TF*_ETI_, and the *SFR* by generating an ablation time series for each possible combination of these parameters and choose the one to minimize the factor *f* as defined below:

8 $$f={\rm{\Sigma }}|\frac{{A}_{{\rm{cum}}}-{A}_{{\rm{obs}}}}{{A}_{{\rm{obs}}}}|$$

Where *A*_cum_ is the cumulated *A*_TI_ or *A*_ETI_, and *A*_obs_ is derived with the sounding height ranger and the density parameterization (Eqs 4 and 5), at a daily time scale. The *TF* values are between 0 and 100 kg m^−2^ °C^−1^ and *SRF* between 0 and 1 kg m^−2^ W^−1^ m^2^ day^−1^. We considered the calibration failed if, to minimize *f*, one of the parameters must take its maximum value, or if all parameters must be 0.

## Supplementary information

Supplementary information

## Data Availability

The datasets generated during and analyzed during the current study are available from the corresponding author on reasonable request.

## References

[1] Lutz AF, Immerzeel WW, Shrestha AB, Bierkens MFP (2014). Consistent increase in High Asia’s runoff due to increasing glacier melt and precipitation. Nat. Clim. Chang..

[2] Shea JM, Immerzeel WW (2016). An assessment of basin-scale glaciological and hydrological sensitivities in the Hindu Kush-Himalaya. Ann. Glaciol..

[3] Mott R, Vionnet V, Grunewald V (2018). The seasonal snow cover dynamics: Review on Wind-Driven Coupling Processes. Front. Earth Sci..

[4] Hock R (2003). Temperature index modelling in mountain areas. J. Hydrol..

[5] Hock R (2005). Glacier melt: A review of processes and their modelling. Prog. Phys. Geogr..

[6] Pellicciotti F (2005). An enhanced temperature-index glacier melt model including the shortwave radiation balance: Development and testing for Haut Glacier d’Arolla, Switzerland. J. Glaciol..

[7] Azam MF (2014). Processes governing the mass balance of Chhota Shigri Glacier (western Himalaya, India) assessed by point-scale surface energy balance measurements. Cryosphere.

[8] Sicart JE, Hock R, Six D (2008). Glacier melt, air temperature, and energy balance in different climates: The Bolivian Tropics, the French Alps, and northern Sweden. J. Geophys. Res..

[9] Kayastha, R. B., Takeuchi, Y., Nakawo, M., Ageta, Y. Practical prediction of ice melting beneath various thickness of debris cover on Khumbu Glacier, Nepal, using a positive degree-day factor. In *Debris Covered Glaciers* 71–151 (IAHS Publ. No. 264, 2000).

[10] Yang W (2011). Summertime surface energy budget and ablation modeling in the ablation zone of a maritime Tibetan glacier. J. Geophys. Res. Atmos..

[11] Mölg T, Maussion F, Yang W, Scherer D (2012). The footprint of Asian monsoon dynamics in the mass and energy balance of a Tibetan glacier. Cryosphere.

[12] Sun, W. *et al*. Ablation modeling and surface energy budget in the ablation zone of Laohugou glacier No. 12, western Qilian mountains, China. *Ann. Glaciol*. **55** (2014).

[13] Collier E (2013). High-resolution interactive modelling of the mountain glacier-atmosphere interface: An application over the Karakoram. Cryosphere.

[14] Huintjes E, Neckel N, Hochschild V, Schneider C (2015). Surface energy and MAss balance at Purogangri ice cap, central Tibetan Plateau, 2001–2011. J. Glaciol..

[15] Wagnon P, Sicart J, Berthier E, Chazarin J (2003). Wintertime high-altitude surface energy balance of a Bolivian glacier, Illimani, 6340 m above sea level. J. Geophys. Res..

[16] Mölg T, Hardy DR (2004). Ablation and associated energy balance of a horizontal glacier surface on Kilimanjaro. J. Geophys. Res. D Atmos..

[17] Sicart JE, Wagnon P, Ribstein P (2005). Atmospheric controls of the heat balance of Zongo Glacier (16°S, Bolivia). J. Geophys. Res..

[18] Wagnon, P. *et al*. Seasonal and annual mass balances of Mera and Pokalde glaciers (Nepal Himalaya) since 2007. *Cryosph*. **7**, 1769–1786 (2013).

[19] Huintjes E (2015). Evaluation of a Coupled Snow and Energy Balance Model for Zhadang Glacier, Tibetan Plateau, Using Glaciological Measurements and Time-Lapse Photography. Arctic, Antarct. Alp. Res..

[20] Carenzo M, Pellicciotti F, Rimkus S, Burlando P (2009). Assessing the transferability and robustness of an enhanced temperature-index glacier-melt model. J. Glaciol..

[21] MacDougall AH, Wheler BA, Flowers GE (2011). A preliminary assessment of glacier melt-model parameter sensitivity and transferability in a dry subarctic environment. Cryosphere.

[22] Ragettli S (2015). Unraveling the hydrology of a Himalayan catchment through integration of high resolution *in situ* data and remote sensing with an advanced simulation model. Adv. Water Resour..

[23] Shea JM (2015). A comparative high-altitude meteorological analysis from three catchments in the Nepalese Himalaya. Int. J. Water Resour. Dev..

[24] Bookhagen, B. & Burbank, D. W. Toward a complete Himalayan hydrological budget: Spatiotemporal distribution of snowmelt and rainfall and their impact on river discharge. *J. Geophys. Res. Earth Surf*. **115** (2010).

[25] Immerzeel WW, Petersen L, Ragettli S, Pellicciotti F (2014). The importance of observed gradients of air temperature and precipitation for modeling runoff from a glacierized watershed in the Nepalese Himalayas. Water Resour. Res..

[26] Stigter EE (2018). The importance of snow sublimation on a Himalayan glacier. Front. Earth Sci..

[27] Adhikary S (2012). Seasonal and spatial variation of solar radiation in Nepal Himalayas. J. Hydrol. Meteorol..

[28] Sherpa SF (2017). Contrasted surface mass balances of debris-free glaciers observed between the southern and the inner parts of the Everest region (2007–15). J. Glaciol..

[29] Plüss C, Ohmura A (1997). Longwave Radiation on Snow-Covered Mountainous Surfaces. J. Appl. Meteorol..

[30] Sicart JE, Pomeroy JW, Essery RLH, Bewley D (2006). Incoming longwave radiation to melting snow: observations, sensitivity and estimation in Northern environments. Hydrol. Process..

[31] Rabatel A (2013). Current state of glaciers in the tropical Andes: a multi-century perspective on glacier evolution and climate change. Cryosph..

[32] Sauter T, Obleitner F (2015). Assessment of the uncertainty of snowpack simulations based on variance decomposition. Geosci. Model Dev. Discuss..

[33] Vionnet V (2014). Simulation of wind-induced snow transport and sublimation in alpine terrain using a fully coupled snowpack/atmosphere model. Cryosph..

[34] Vionnet V (2017). High-Resolution Large Eddy Simulation of Snow Accumulation in Alpine Terrain. J. Geophys. Res. Atmos..

[35] Gerber F (2018). Spatial variability in snow precipitation and accumulation in COSMO–WRF simulations and radar estimations over complex terrain. Cryosph..

[36] Baral P (2014). Preliminary results of mass-balance observations of Yala Glacier and analysis of temperature and precipitation gradients in Langtang Valley, Nepal. Ann. Glaciol..

[37] Litt M, Sicart JE, Helgason W (2015). A study of turbulent fluxes and their measurement errors for different wind regimes over the tropical Zongo Glacier (16°S) during the dry season. Atmos. Meas. Tech..

[38] Pellicciotti F (2008). A study of the energy balance and melt regime on Juncal Norte Glacier, semi-arid Andes of central Chile, using melt models of different complexity. Hydrol. Process..

[39] Kayastha RB, Ohata T, Ageta Y (1999). Application of a mass-balance model to a Himalayan glacier. J. Glaciol..

[40] Yang W (2013). Mass balance of a maritime glacier on the southeast Tibetan Plateau and its climatic sensitivity. J. Geophys. Res. Atmos..

