# Supplementary material for: Glacier ablation and temperature indexed melt models in the Nepalese Himalaya
Source: Sci Rep. 2019 Mar 27;9:5264. doi: 10.1038/s41598-019-41657-5 (PMC6437175; doi:10.1038/s41598-019-41657-5)
Supplement: Supplementary file 1 — Supplementary information [file 41598_2019_41657_MOESM1_ESM.pdf]

Supplementary Information, for:

Glacier ablation and temperature indexed melt models in the Nepalese Himalaya.

Maxime Litt*[1,2], Joseph Shea[3], Patrick Wagnon[4], Jakob Steiner[1], Inka Koch[2], Emmy Stigter[1], Walter Immerzeel[1]

1) Physical Geography, Faculty of Geosciences, Utrecht University, Utrecht, The Netherlands.
2) International Center for Integrated Mountain Development, Kathmandu, Nepal.
3) Geography Program, University of Northern British Columbia, Prince George, Canada.
4) Institut des Geosciences de l'Environnement, Université Grenoble-Alpes, Grenoble, France.

Figure captions

Figure S1: Surface energy balance (black thick lines) and decomposition into its daily components. Surface warming (resp. cooling) contributions are stacked such as the net positive (resp. negative) contribution is the border line of the highest area (resp. lower). Daily mean surface energy balance is shown with the black line. The daily mean T_a is shown with the purple line.

Figure S2: Ablation factors obtained by calibrating the temperature index and the enhanced temperature index models with data from Mera Glacier, 5360 m a.s.l., in 2014 and 2015 and Yala Glacier, 5330 m a.s.l., in 2014, 2016 and 2017. Panels a, b, c and d show the results obtained when combining all data available for the indicated year. Panels e, f, g and h show the results obtained using only the monsoon data.

Figure S3: The density parameterization used in this study, compared to on-field measurements.

Tables captions

Table S1: Correlation coefficients between SEB and each of its component, and air temperature and albedo, during different seasons.

Table S2: Contribution to correlation coefficients between SEB and each of its component, between SEB and air temperature, and between SEB and albedo.

Table S3: Melt and sublimation contribution to the overall sum of both.

Table S4: Ablation factors obtained by calibrating the temperature index and the enhanced temperature-index models. Empty values indicate unavailable data or failure of at optimizing the model.

Figures

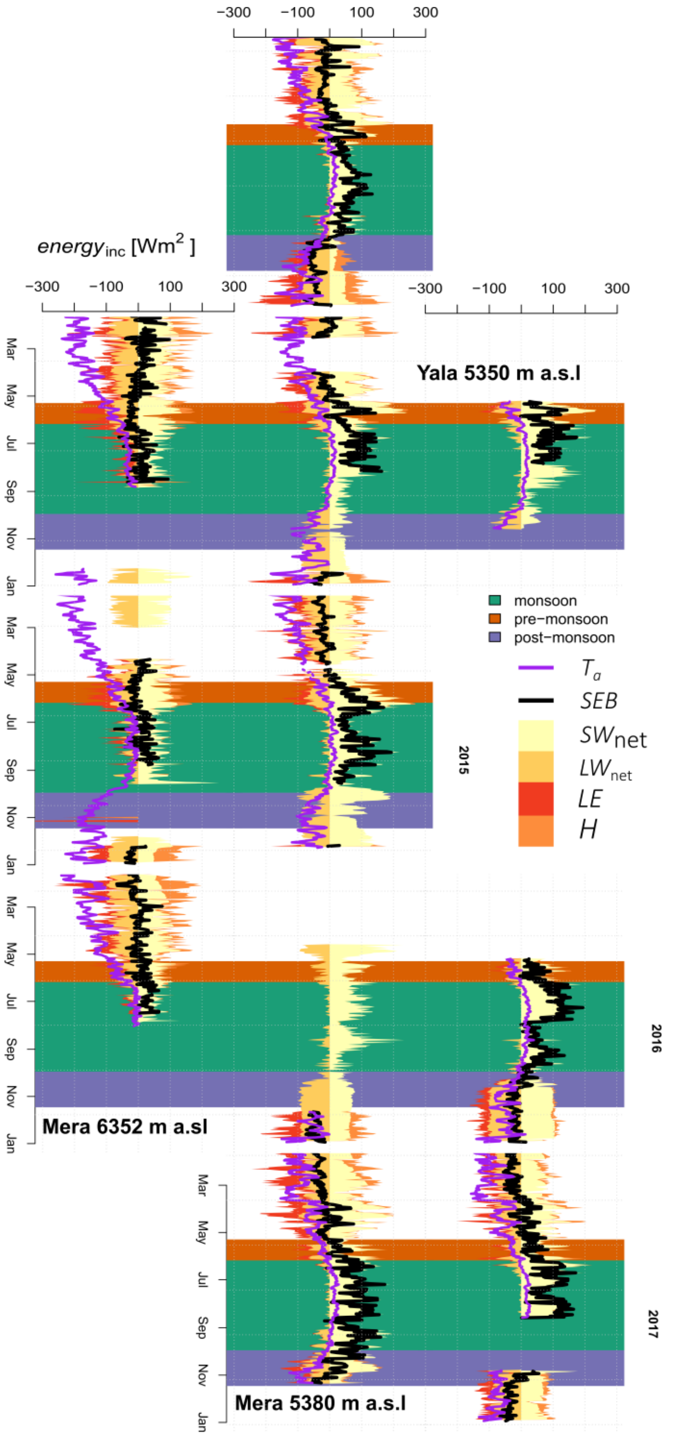

Figure S1: Surface energy balance (black thick lines) and decomposition into its daily components. Surface warming (resp. cooling) contributions are stacked such as the net positive (resp. negative) contribution is the border line of the highest area (resp. lower). Daily mean surface energy balance is shown with the black line. The daily mean T_a is shown with the purple line.

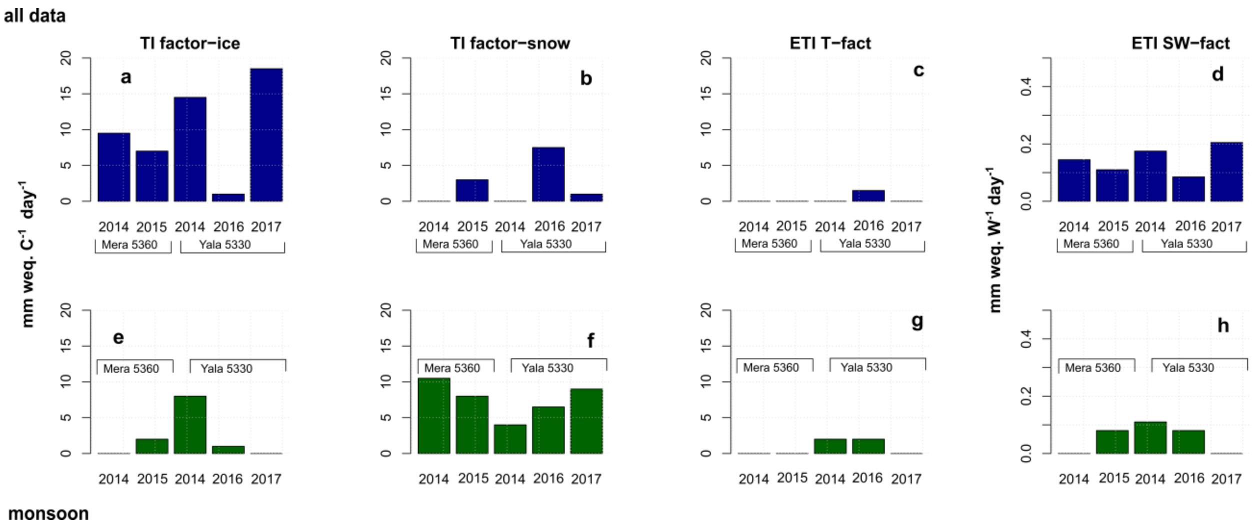

Figure S2: Ablation factors obtained by calibrating the temperature index and the enhanced temperature index models with data from Mera Glacier, 5360 m a.s.l., in 2014 and 2015 and Yala Glacier, 5330 m a.s.l., in 2014, 2016 and 2017. Panels a, b, c and d show the results obtained when combining all data available for the indicated year. Panels e, f, g and h show the results obtained using only the monsoon data.

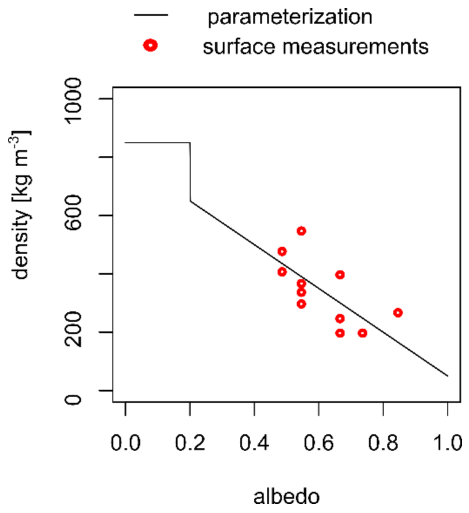

Figure S3: The density parameterization used in this study, compared to on-field measurements.

Tables

Table S1: Correlation coefficients between SEB and each of its component, and air temperature and albedo, during different seasons.

Site	season	year	T_a	SW_{net}	albedo	LW_{net}	R_{net}	H	LE
Mera Glacier, 5380 ma.s.l., ablation zone	pre-monsoon	2013	-	0.72	-0.78	-0.06	0.93	0.32	0.55
	monsoon		-	0.81	-0.84	0.25	0.98	-0.07	0.45
	post-monsoon		-	-0.37	0.05	0.89	0.86	-0.71	0.84
	pre-monsoon	2014	0.23	0.89	-0.83	-0.65	0.93	0.09	-0.30
	monsoon		0.63	0.87	-0.92	0.02	1,00	-0.12	0.21
	post-monsoon		-	-	-	-	-	-	-
	pre-monsoon	2015	0.63	0.87	-0.82	-0.55	0.93	0.46	-0.04
	monsoon		0.51	0.93	-0.92	-0.42	0.99	0.24	-0.30
	post-monsoon		-	-	-	-	-	-	-
	pre-monsoon	2016	-	-	-	-	-	-	-
	monsoon		-	-	-	-	-	-	-
	post-monsoon		-	-	-	-	-	-	-
	pre-monsoon	2017	-	0.74	-0.53	-0.23	0.94	0.50	-0.33
	monsoon		-	0.93	-0.81	-0.19	1,00	0.05	0.01
	post-monsoon		-	0.69	-0.12	0.37	0.95	-0.50	0.39
Yala Glacier, 5330 m a.s.l., ablation zone	pre-monsoon	2016	0.58	0.78	-0.74	-0.29	0.93	0.54	0.15
	monsoon		0.59	0.95	-0.91	0.09	1,00	0.25	0.32
	post-monsoon		0.51	-0.39	0.35	0.69	0.74	-0.42	0.82
	pre-monsoon	2017	0.52	0.87	-0.80	-0.60	0.95	0.32	-0.27
	monsoon		0.52	0.93	-0.73	-0.07	1,00	-0.05	-0.05
	post-monsoon		-0.62	0.64	-0.56	0.68	0.93	-0.56	0.50
	pre-monsoon	2014	0.40	0.84	-0.79	-0.41	0.94	0.16	0.00
	monsoon		0.22	0.92	-0.68	-0.35	1,00	-0.02	-0.10
	post-monsoon		0.00	-	-	-	-	-	-
Mera Glacier, 6542 m a.s.l., accumulation zone	pre-monsoon	2014	-0.02	0.15	-0.15	-0.10	0.26	-0.01	0.27
	monsoon		0.26	0.10	-0.17	0.38	0.67	-0.01	0.72
	post-monsoon		-	-	-	-	-	-	-
	pre-monsoon	2015	0.13	0.05	-0.06	-0.07	-0.06	0.11	0.57
	monsoon		0.25	-0.10	0.01	0.38	0.47	0.12	0.78
	post-monsoon		-	-	-	-	-	-	-
	pre-monsoon	2016	-0.21	0.17	-0.41	-0.18	0.00	0.41	0.69
	monsoon		0.10	0.45	-0.12	0.05	0.70	0.28	0.57
	post-monsoon		-	-	-	-	-	-	-

Table S2: Contribution of each SEB's component variability and of albedo, to the correlation coefficients between SEB and air temperature and to the correlation between SEB and SW_{net}.

$R(T_a, SEB)_x$		SW_{net}	albedo	LW_{net}	H	LE
Yala, 5330 m a.s.l., ablation zone.	pre-monsoon	+0.34	+0.00	-0.17	+0.13	+0.05
	monsoon	+0.49	-0.01	+0.10	-0.00	+0.04
	post-monsoon	+0.08	-1.87	+0.04	+0.07	+0.13
Mera 5360 m a.s.l., ablation zone.	pre-monsoon	+0.42	-0.00	-0.20	+0.13	+0.04
	monsoon	+0.58	-0.00	0.03	-0.00	+0.03
	post-monsoon	+0.28	-2.55	-0.05	NA	NA
Mera Glacier, 6542 m a.s.l., accumulation zone.	pre-monsoon	+0.31	-0.00	-0.15	+0.08	-0.00
	monsoon	+0.37	-0.00	0.09	-0.01	0.11
	post-monsoon	NA	NA	NA	NA	NA

$R(SW_{net}, SEB)_x$		albedo	LW_{net}	H	LE
Yala, 5330 m a.s.l., ablation zone.	pre-monsoon	-0.00	-0.56	+0.07	-0.21
	monsoon	-0.00	-0.17	+0.01	-0.02
	post-monsoon	-0.62	-0.32	-0.20	+0.00
Mera 5360 m a.s.l., ablation zone.	pre-monsoon	-0.00	-0.53	+0.08	-0.20
	monsoon	-0.00	-0.22	+0.02	-0.04
	post-monsoon	-0.74	-0.24	-0.24	+0.10
Mera Glacier, 6542 m a.s.l., accumulation zone.	pre-monsoon	-0.00	-0.45	+0.06	-0.16
	monsoon	-0.00	-0.15	+0.02	+0.00
	post-monsoon	NA	NA	NA	NA

Table S3: Melt and sublimation contribution to the overall sum of both.

		**mean daily melt from SEB, kg m^{-2}**	**mean daily sublimation kg m^{-2}**	**sum of the two**	**percentage contribution**		**number of days**
Mera 5360 m a.s.l., ablation zone.	pre-monsoon	47.8	-3.3	51.0	0.9363	0.0637	109
	monsoon	78.9	-0.4	7.9	0.9944	0.0055	299
	post-monsoon	6.6	-1.9	8.5	0.7731	0.2269	81
Yala, 5330 m a.s.l., ablation zone.	pre-monsoon	31.5	-1.8	33.3	0.9453	0.0547	64
	monsoon	68.5	-0.1	68.5	0.9987	0.0013	164
	post-monsoon	1.5	-2.4	3.9	0.3844	0.6156	37
Mera Glacier, 6542 m a.s.l., accumulation zone.	pre-monsoon	0.0	-7.1	7.1	0.0000	1,0000	42
	monsoon	12.5	-8.8	21.3	0.5883	0.4117	64
	post-monsoon	-	-	-	-	-	-

Table S4: Ablation factors obtained by calibrating the temperature index and the enhanced temperature-index models. Empty values indicate unavailable data or failure of at optimizing the model.

Site	Year	Season	TF_{ETI} mm w.equ C^{-1}	SRF mm we W^{-1} m^2	TF (Ice) mm we C^{-1}	TF (Snow) mm we C^{-1}	Days of data available.
Mera 5360 m a.s.l., ablation zone.	2014	All	0.0	0.1	0.0	9.5	185
		Pre-mon.	6.0	0.2	45.0	25.5	26
		monsoon	0.0	0.0	10.5	0.0	61
		Post-mon.	0.0	0.0	10.5	0.0	0
	2015	All	0.0	0.1	3.0	7.0	246
		Pre-mon.	13.5	0.1	0.0	36.5	26
		monsoon	0.0	0.1	8.0	2.0	104
		Post-mon.	-	-	-	-	0
	2016	All	-	-	-	-	193
		Pre-mon.	-	-	-	-	26
		monsoon	-	-	-	-	112
		Post-mon.	-	-	-	-	29
Yala, 5330 m a.s.l., ablation zone.	2014	All	0.0	0.2	0.0	14.5	81
		Pre-mon.	0.0	0.3	-	-	26
		monsoon	2.0	0.1	4.0	8.0	52
		Post-mon.	-	-	-	-	0
	2016	All	1.5	0.1	7.5	1.0	220
		Pre-mon.	0.0	0.1	-	-	26
		monsoon	2.0	0.1	6.5	1.0	105
		Post-mon.	0.0	0.1	-	-	38
	2017	All	0.0	0.2	1.0	18.5	282
		Pre-mon.	0.0	0.2	-	-	26
		monsoon	-	-	9.0	0.0	71
		Post-mon.	-	-	-	-	17
Mera Glacier, 6542 m a.s.l., accumulation zone.	2014	All	-	-	-	-	183
		Pre-mon.	-	-	-	-	26
		monsoon	-	-	-	-	67
		Post-mon.	-	-	-	-	0
	2015	All	14.5	0.0	-	-	50
		Pre-mon.	-	-	-	-	17
		monsoon	2.5	0.0	-	-	5
		Post-mon.	-	-	-	-	0
	2016	All	0.0	0.3	-	-	122
		Pre-mon.	-	-	-	-	8
		monsoon	-	-	-	-	19
		Post-mon.	-	-	-	-	0
